# Factors affecting production of competent health workforce in Tanzanian health training institutions: a cross sectional study

**DOI:** 10.1186/s12909-022-03719-7

**Published:** 2022-09-05

**Authors:** A Nyamtema, GM Karuguru, AS Mwangomale, AF Monyo, E Malongoza, P Kinemo

**Affiliations:** 1 Tanzanian Training Centre for International Health, Ifakara, Tanzania; 2 St. Francis University College for Health and Allied Sciences, Ifakara, Tanzania; 3 National Council for Technical Education, Dar es Salaam, Tanzania; 4 Sikika, Dar es Salaam, Tanzania

**Keywords:** Health training institutions, Clinical medicine, Instructor-students ratio, Clinical competency deficits

## Abstract

**Background:**

In 2008, the government of Tanzania adopted a competency-based education and training (CBET) system to improve medical training. Yet there are still frequent observations of competency deficits among graduates, suggesting that the goal has not sufficiently been met. This study was designed to assess the underlying context of competency deficits in the health workforce in Tanzania and to provide recommendations for improvement.

**Methods:**

A cross-sectional study using document analysis and focus groups was carried out in 13 training institutions that provided a diploma course in clinical medicine. The research team assessed availability and adequacy of instructors, physical resources and the process and systemic factors that impact curriculum implementation outcomes.

**Results:**

Six (46%) institutions had 75% or more of their teaching staff not trained in curriculum delivery and instructional methods. Seven (54%) institutions had lower instructor-students ratio than recommended (1:25). Overall, the full-time instructors in all institutions constituted only 44% of the teaching staff. Although all institutions had an adequate number of classrooms, the rooms were of small size with dilapidated walls, and had inadequate number of desks/ seats for students. Clinical skills laboratories existed in 11 (85%) institutions, but the majority were of small size, and were not fully equipped as per guidelines and were rarely used. Libraries were available in 12 (92%) institutions but five had seating capacities of 10% or less of the available students. Participants of focus group discussion in the majority of the institutions reported inadequate time allocated for practice and support from the clinical instructors at the practicum sites. Six (46%) institutions had no functioning governing/advisory boards and five (38%) lacked quality assurance policies and implementation plans.

**Conclusions:**

Currently, health-training institutions in Tanzania are ill-equipped to produce competent clinicians because of major gaps in the structural, process and systemic components. These findings call for major investment to facilitate production of a competent health workforce.

## Introduction

Inadequate training background is a root cause of competency deficits of the health workforce [1, 2]. Studies show that many health training institutions (HTIs) in sub-Saharan Africa have failed to meet the required training standards, and therefore have been offering sub-standard training programmes which culminate in a producing less competent health workforce thus weakening the quality of care [3–5]. Competency deficits of the health workforce is a significant cause of medical errors which are associated with adverse impacts on patient safety and health economics. Competency deficits are associated with diagnostic error or delay in diagnosis, treatment and preventive errors. Diagnostic and treatment errors lead to no or incorrect treatment and can worsen a patient’s condition or even cause death[1]. Annually, 421 million hospitalizations take place worldwide, from those an estimation of around 43 million medical errors occur. Low-middle income countries account for two-thirds of these medical errors [6]. It is estimated that medical errors account for as many as 251,000 deaths annually in the United States (U.S)., making medical errors the third leading cause of death [7]. A prospective cohort study done in three regions in Tanzania from 2010—2014 indicated that 45% of maternal deaths and 66% of intrapartum and early neonatal deaths were attributed to inadequate skills of health care providers [8]. In 2014, it was reported that health care providers in Tanzania could only correctly diagnose 60% of common conditions, ranging from 70% in the urban areas down to 44% in public facilities located in rural areas [3]. Medical errors cost the UK over £2 billion in 2000 and the US $19.5 billion in 2008 [9, 10]. Although the economic impact of medical errors has not been well studied in Tanzania, it is expected to be high and may include additional medical cost, lost productivity from missed work and repeated training [9, 11].

The determinants of the competence of graduates from HTIs can be classified into structural, process and systemic components [12, 13]. In this study the term “structure” means resources i.e., the condition under which teaching and learning processes are organized and support the provision of professional experiences to students such as premises, buildings, equipment, staff, curriculum, laboratories and practicum sites; while “process” means the activities that constitute the teacher-learner interactions including instructional and assessment methods, and proper utilization of the structures. On the other hand, “systemic components” mean interactions of different items and activities which include the organization structure and levels of responsibilities such as advisory or governing boards, institutional governance and administration, students’ welfare, staff development plans and motivational strategies. This study was designed to assess the capabilities of HTIs to produce competent graduates of the ordinary diploma in clinical medicine.

## Methods and tools

### Design and setting of the study

At the time of designing the study there were 157 registered HTIs (universities and technical institutions) in a projected Tanzanian population of 60 million, which offered various training programs for health workers. Of these, 26 (17%) were technical clinical medicine training institutions. Of the training institutions for clinical medicine, 20 (77%) were public. A cross-sectional study with quantitative and qualitative data collection methods was conducted in 2018. We used the STROBE cross sectional checklist when writing our report [14].

### Sampling technique and size

HTIs were stratified based on geographical locations (health zones). In Tanzania there are eight health zones: Eastern, Western, Lake, Central, South-Western, Northern, Southern Highland and Southern zones. This was followed by stratifying the HTIs based on ownership (public, FBO, private and public–private institutions). Purposeful sampling was performed to ensure that all zones and a few available private and public–private HTIs were represented in this study to get a representative picture of the underlying context. Thirteen HTIs that implemented the competency-based education and training (CBET) curriculum for clinical medicine for at least 3 years were selected for the study. The sample size thus constituted 50% of the HTIs providing an ordinary diploma in clinical medicine in Tanzania. The distribution included six public institutions, five faith-based organizations (FBO), one public–private partnership and one private institution.

### Data collection process

The scope of assessment included the human resources, infrastructure and learning resources, governance and administration, and curriculum implementation at the HTIs. Assessment of human resources included the number and categories of full-time and part-time teaching staff based on the national guidelines [15], the number of tutors who left the job and those recruited between 2016 and 2017, and the number of teaching staff trained in curriculum delivery methods. These data were accessed from the principals’ offices.

Infrastructure and learning resources included the number of students, programs at each institution, existence of classrooms, library, computer laboratory, skills laboratory, reliable power supply, availability and quality of classroom furniture, teaching/facilitating equipment and gadgets and existence of reliable internet connectivity. While the number of students was accessed from the principals’ offices, infrastructure and learning resources were verified by the research team.

For the governance and administration the team assessed the existence and functioning of the governing or advisory board verified by documented proceedings, organogram and lines of responsibilities. The existence of institutional policies including the quality assurance policy and implementation plan, regulations, procedures and strategies to guide implementation of the planned objectives were also assessed and verified.

Curriculum implementation: instructional methods and assessment were assessed by verifying the existence or availability of an implementation plan (theory and practical rotation master-plan and module delivery schedule), module delivery plans, oversight mechanism, practical log book; academic committee and examination oversight, assessment plans for all modules, evidence on utilization of assessment plans, examination regulations, reports prepared by examination setters, moderators, and markers, procedures for declaring examination results board’s approval of examination results and external examiner’s reports.

### Qualitative data collection

One focus group (FG) for discussion was conducted in each training institution. Each group was made of 8 students in second and third year of studies including at least two student leaders. Second and third year students were purposively selected because they were believed to have the potential to provide rich, relevant and diverse data pertinent to the research question. A moderator and research assistant guided the discussion, took notes and recorded the discussion. The discussion focused on the adequacy and availability of essential equipment and infrastructure to support teaching and learning processes; adequacy of faculty; commitments of the teaching staff and clinical instructors to the teaching role; capacity and effectiveness of the practicum site; and mechanisms in place for the assessment of teaching and learning processes. Participants were encouraged to openly convey their viewpoints using questions and prompts. Each thematic area was discussed until no new relevant information was being obtained from any participant. Focus group discussions lasted for around 40 min.

### Data management and analysis

All quantitative data, derived from document analysis and direct information provided to the research team were entered directly into Microsoft excel for analysis. Results were presented in terms of instructor-students ratio, staff disposition, existence and equipment of the instruction rooms, applied clinical laboratories, skills laboratories and information resource centres. These parameters were compared with the national standards which were developed in conformity with the National Council for Technical Education (NACTE) accreditation standards and the Ministry of Health for HTIs [12, 15]. Qualitative data was analysed using content analysis methodology as described by Graneheim and Lundman [16].

## Results

### Educational programs, students and human resources

All health-training institutions provided an ordinary diploma in clinical medicine, nine community health program, two pharmaceutical sciences, five nursing & midwifery and four medical laboratory sciences. The number of students taking an ordinary diploma course in clinical medicine per institution ranged from 62 to 430. Public institutions tended to have fewer educational programs than the faith-based organizations (Table 1). Almost half (46%) of the training institutions had over three quarters (75%) of the teaching staff not trained in curriculum delivery methods. Among these, four i.e., two thirds (67%) were public health institutions. Seven (54%) institutions had lower instructor-students ratio than recommended (1:25) by the regulatory bodies. The instructor-to-students ratio was as low as 1:67 at Lindi Clinical Officer Training Centre (COTC).

**Table 1:** Instructor-students ratio and the proportions of the teaching staff trained in curriculum delivery methods

Names of HTIs	Number of training programs	Total students (all programs)	Full-time teaching staff	Total teaching staff (full & part time)	Total trained in curriculum delivery methods (full & part time) n (%)	Instructor-students ratio	Attrition rate
Mtwara COTC*	3	798	13	41	24 (59%)	1:61	0%
St. Augustine HTI	3	202	11	19	15 (79%)	1:18	24%
Mvumi Inst. HS	4	418	24	45	8 (18%)	1:17	48%
Nkinga Inst. HS	4	494	18	29	9 (31%)	1:27	46%
TTCIH^†^	1	181	15	41	34 (83%)	1:12	0%
Mafinga COTC*	2	300	5	33	2 (6%)	1:60	0%
Bumbuli COTC	2	480	9	25	17 (68%)	1:53	16%
St. Aggrey CHS	5	617	16	33	7 (21%)	1:39	50%
Mbeya CHS*	1	200	16	26	17 (65%)	1:13	0%
Lindi COTC*	1	400	6	24	3 (13%)	1:67	0%
Masasi COTC*	1	238	10	25	2 (8%)	1:24	12%
Kilosa COTC*	2	259	7	22	4 (18%)	1:37	46%
Kolandoto CHS	4	402	25	32	18 (56%)	1:16	36%
Overall	-	4989	175	395	160 (41%)	1:29	26%

Note: *COTC* Clinical officer training centre, *HTI* Health training institution, *CHS* Centre of health sciences, *HS* Health sciences, *TTCIH* Tanzanian training centre for international health

*Public HTI

^†^Public private partnership HTI

Overall, full-time instructors constituted only 44% of the teaching staff across all institutions. The proportion of full-time instructors was less than one third of the teaching staff in four (31%) institutions. The overall employees' turnover for the three-year period (2016—2018) was 26%. The rate was as high as 46% in four training institutions. The main reason for leaving their jobs in the private and FBO institutions was related to new employment in the public sector while in public institutions reasons were either transfer or retirement. HTIs were affected by inadequate staff numbers, regular absenteeism and inadequate commitments of the teaching staff.

“The number of instructors is inadequate taking into consideration the number of subjects and educational programs offered in this college. One instructor teaches all clinical officers’ classes from first to third year.” ( FG participant, HTI 12).

“Instructors are very few, until now there are only two doctors who are not available at the college most of the time … we are compelled to rely on ourselves, we take our learning modules and read ourselves, we browse the internet and go to the hospital and ask questions whenever we do not get support from the college.” (FG participant, HTI 10).

The majority of the training institutions hired part-time teachers from the teaching hospitals to bridge the gaps. Those teachers could not properly address these gaps, because they were usually heavily occupied with their primary responsibilities in their work places, particularly with attending patients*.* Some institutions hired very inexperienced staff like doctors still in the internship training program. Even in the institutions where the number was considered to be adequate, instructors were commonly not available, for various reasons including government assignments elsewhere.

### Classrooms

All institutions had an adequate number of classrooms for the clinical medicine program but these were usually not well equipped. Ten institutions (77%) had a shortage of complete sets of chairs & desks in the classrooms. Almost one third (31%) of the institutions had serious deficits in sets of chairs and desks in the classrooms ranging from 35 to 68%. Classrooms in the majority of the training institutions were of small size, lacked electricity, and had cracks and old paint coming off the walls, an inadequate number of desks and seats (chairs).

“The walls of the classrooms have cracks and the ceiling fans do not function at all because they are very old … students are at risk of accidents when they are in the classrooms” (FG participant, HTI 11).

“Class sessions (lectures) are conducted in shifts, one after the other, because the number of desks and seats are inadequate.” (FG participant, HTI 06).

Quite often teachers postponed lecture-sessions for reasons related to inadequate number of classrooms and/ or teaching aids like projectors till when another teacher finishes a session for the other class.

“Although the classrooms are available, the number and size are inadequate when compared with the available number of students for all programs.” (FG participant, HTI 04).

“(…) Classrooms are inadequate, our college has many students … chairs and desks are greatly inadequate to the extent that students are compelled to keep chairs otherwise they do not get anywhere to sit during the lectures.” (FG participant, HTI 08). Similar statements were also given by FG participants at HTI 13.

### Clinical skills laboratories and libraries

One institution had no clinical skills laboratory. Only one institution had a clinical skills laboratory with complete sets of basic equipment for the surgery, paediatrics, internal medicine and obstetrics/gynaecology sub-sections. Other institutions had incomplete sets of basic equipment in one or more of these sub-sections (Table 2). The FG participants from approximately half of the institutions reported that the clinical skills laboratories were either small in size or not adequately used for teaching and learning purposes.

“A clinical laboratory exists, but it is very small. We are compelled to work in groups of 10 to 15 while the class has ninety students”, (FG participant, HTI 11).

“Although our college has a clinical skills laboratory, we do not use it, and the mannequins are not used at all”, (FG participant, HTI 01).

**Table 2:** Proportions of training institutions with basic learning tools (equipment) in the clinical skills laboratories (*n* = 13)

Basic learning tools (equipment)	Complete set	Incomplete set	None of the equipment exists
Hand washing facilities	69	23	8
Screens	62	31	8
Examination beds	46	38	15
Surgery sub-section with basic surgical kits	38	23	46
Medicine sub-section: BP machine, stethoscope, patellar hammer etc	69	15	15
Paediatrics sub-section: neonatal & resuscitation kit	38	38	23
Obstetrics/Gynaecology sub-section: obstetric mannequins, delivery mannequins, MVA set, vacuum extractors	38	23	38

Note: *MVA* Manual vacuum aspiration

Eleven (85%) institutions had a library with a fairly adequate number of relevant books for the clinical medicine program, ranging from 201 to 60,000 books (Table 3). Five institutions had libraries with seating capacities for 10% or less of the available students for all programs. Libraries in eight (61%) institutions had no internet connectivity. More than one third (38%) of the institutions had no access to e-books.

**Table 3:** The capacity and equipment of the information resource centres (libraries)

	Books for all programs	Relevant books for CO program	Manuals and Journals	Functioning computers	Seating capacity of the library	Internet connectivity	e-books	Librarians
Mtwara COTC	72,000	60,000	30	30	100	YES	1	1
St. Augustine HTI	2,100	201	0	0	60	NO	50	2
Mvumi Inst. HS	2,300	900	100	55	90	YES	200	1
Nkinga Inst. HS	1,392	1,392	300	30	50	YES	150	1
TTCIH	6,000	6,000	1,500	20	30	YES	4,000	2
Mafinga COTC	2,600	2,000	100	12	30	NO	50	1
Bumbuli COTC	2,422	2,179	0	28	50	NO	0	2
St. Aggrey CHS	1,200	50	0	36	58	NO	1,500	2
Mbeya CHS	3,939	695	75	0	0	NO	0	1
Lindi COTC	533	433	100	10	20	NO	0	0
Masasi COTC	360	360	0	25	40	NO	0	1
Kilosa COTC	250	60	0	12	0	NO	0	0
Kolandoto CHS	2,226	1,500	600	15	100	YES	3,000	1

Note: *COTC* Clinical officer training centre, *HTI* Health training institution, *CHS* Centre of health sciences

Based on the FG participants’ opinions the information resource centres were generally small in size, short of relevant books for the program of clinical medicine and inadequately used because were usually opened during the class hours only. Because of the limited number of librarians in most institutions, libraries were opened only during the class hours, making it difficult for students to use them. In addition, students were often not allowed to borrow relevant books because of their scarcity.

Although computer laboratories were available in more than three quarters (77%) of the HTIs, the majority were small with an inadequate number of functioning computers and lacked internet connectivity.

“There is a computer laboratory with several computers, but only five of them are functioning … For that reason students use their own computers.” (FG participant, HTI 12).

### Clinical practicum sites

All practicum sites (teaching hospitals) had departments for general surgery, obstetrics & gynaecology, paediatrics and internal medicine. Ten (77%) institutions also had sites for surgical specialties (ear, nose and throat [ENT], dental & ophthalmology). Overall, FG participants from the majority of the institutions reported that the practice sites were sufficient for teaching and learning purposes and that students were encouraged by their teachers to go there. However, they also reported some shortfalls, including inadequate essential supplies like gloves, too brief rotations and inadequate support from the clinical instructors. Some hospitals required the HTI to supply gloves for students to use when assisting and performing clinical procedures. The HTIs however supplied very few gloves, which limited students' opportunities to assist and perform an adequate number of procedures.

### Governance and administration, and quality assurance policy

Six (46%) HTIs did not have functioning governing or advisory boards (Table 4). Only 10 (77%) had strategic plans and governing policies and procedures. Five (38%) did not have quality assurance policies or implementation plans.

**Table 4:** Proportion of HTIs with academic calendar, teaching timetable, teaching evaluation forms, examination regulations and approval by the governing or advisory board (*n* = 13)

	%
Institutional academic calendar and teaching timetable	85
Teaching evaluation forms	85
Students practical log books	100
Examination regulations	100
Institutions conducting assessment as per NACTE* procedures	100
Examinations committee exists with minutes of the meetings	100
Records of continuous assessment and end of semester examination results	100
Examination results are approved by governing or advisory board	54

^*^NACTE National council for technical education

Participants in the FG noted that their institutions had mechanisms in place to assess learning and teaching processes including the use of procedural logbooks for students, examinations and approvals, administration of the teaching assessment forms by students as well as classroom attendance registers for students and teachers. However, in some institutions, students were not involved in evaluating the teaching process.

## Discussion

The main objective of a medical curriculum is to produce a competent health workforce, i.e. to provide medical students with knowledge, skills and attitudes required for their practice. This study has revealed serious structural, process and systemic gaps, arguably three components that are essential for quality program delivery in HTIs.

### Instructors and skills laboratories

The medical profession requires knowledge and psychomotor skills that are dependent on instruction, training, and ongoing practice; only then is it possible to develop and maintain medical competence. Skilled instructors play a pivotal role in producing a competent health workforce through proper teaching, assessment and nurturing both theoretically and practically [17–19]. Adequate instructor-student ratios are imperative for imparting knowledge and skills that lead to clinical competence. Most published curricular requirements for psychomotor skills training involving direct physical supervision by an instructor include a minimum instructor-to-student ratio of 1:5 to 1:10, depending on the skill being taught [20–22]. The low instructor-students ratio, combined with a small proportion of the teaching staff trained in curriculum delivery and instructional methods suggests a worrisome state of the capability of Tanzanian HTIs to produce a competent health workforce. In these institutions skills laboratories were ill-equipped and sub-optimally utilized for teaching and learning processes. Clinical skills laboratories are essential teaching tools that should be integrated by HTIs into their curricula delivery processes. Clinical skills laboratories help to ensure that all students acquire the necessary knowledge and clinical skills, and are properly assessed before practicing on real patients [23–25]. In order to simulate learning in a reflective manner, clinical skills laboratories should be appropriately equipped and in a conducive environment, suitable to support teaching and learning processes. Adequate and appropriate time should also be availed for students to frequently practise all necessary hands-on skills relevant to clinical practice.

### Classrooms and libraries

Although all training institutions had an adequate number of classrooms, they were small, poorly equipped with teaching aids, lacking electricity and internet access, with dilapidated walls, and few desks and seats for students. Studies indicate that conducive physical atmosphere of the classroom can help motivate, promote and improve learning [26]. Inadequate number of chairs for students in the classrooms found in these institutions may imply an unsupportive and demotivating learning environment that may be associated with failure of the students to pay attention and may contribute to absconding the teaching sessions [27]. These findings suggest the need to improve and equip the existing classrooms to create an effective learning environment.

Although libraries existed in almost all (92%) institutions, they were reported to be small in size, with a shortage of prescribed books for the program of clinical medicine and only opened during class hours. In view of these findings, the current libraries do not adequately serve their roles in supporting teaching and learning. Libraries serve crucial roles in learning by supporting appropriate accessibility and utilization of relevant information and providing learning resources for teachers and students [28]. If the library is to serve its role, it must be accessible, have adequate space and must have sufficient relevant and up-to-date learning resources.

### Clinical practice sites

Teaching hospitals play a crucial role in building and strengthening clinical competencies of medical students. They enable students to apply, in real patients’ setting, the specific knowledge and skills learned in the classroom and skills laboratories. To provide adequate experience to students, teaching hospitals must have appropriate and adequate facilities and supportive clinical instructors. Competent and committed clinical instructors play crucial roles in enhancing students’ learning in clinical settings through guiding, modelling, directing, and evaluating student learning. They assist students to integrate theory and practice, promote both professional ethical competence and technical capabilities [29]. The brief time spent in clinical practice sites, combined with inadequate commitment and support from the clinical instructors, as reported by FG participants, may hinder the production of a competent health care workforce.

As has been reported in other studies, our investigation indicates that education in clinical settings competes with patient care, mostly to the disadvantage of the training of clinical skills and competencies [18, 30]. Clinical instructors hired from the teaching hospitals faced the challenging task of delivering quality patient care and facilitating learning at the same time. Similarly, in a number of sites, care providers other than clinical instructors did not support medical students well enough. These findings suggest the need to create an institutional culture in which teaching is regarded as important as patient care. All care providers working in teaching hospitals should support the teaching and learning processes. They should know that teaching hospitals not only act as a hub to treat patients, but are also centres enhancing the production of competent health care providers who will be able to serve in health facilities throughout the country.

### Governance and administration

According to NACTE accreditation standards, Governing and Advisory Boards are key organs responsible for the quality and integrity of the training institution. The absence of these organs in 46% of the institutions leads to sub-optimal educational monitoring and control systems in the management of institutions. Governing and Advisory Boards, should, among others,1) monitor the overall performance of the training institution to ensure that institutional vision and mission are achieved; 2) ensure that institutional practices are consistent with the national regulatory bodies’ guidelines and standards; and 3) ensure that training institutions have functional quality assurance mechanisms. Based on our findings, we suggest that all training institutions form these organs to strengthen the institutional integrity and improve quality assurance.

### Quality assurance policy and implementation plan

The quality assurance policy and implementation plan describe the quality management systems in place that ensure quality of the educational program being delivered. Quality assurance at the training institution encompasses the quality of training and learning activities, availability of appropriate information resources, facilities and functional equipment as well as financial control and sustainability [12]. The absence of quality assurance policies and implementation plans strongly suggests a lack of insight and commitment to quality offerings that could lead to ineffectiveness of the HTI in producing competent graduates. This is a deficiency in institutional organization, management, teaching and learning processes found in our study.

### Limitations

Focus group discussions only involved students. Further studies should also involve other key stakeholders including instructors, HTI management, owners of the HTIs and regulatory authorities (the Ministry of Health and National Council for Technical Education).

## Conclusions

This study has provided a snapshot illustrating the gaps of the infrastructure, human resources, information and learning resources, the process and systemic components in training institutions, which offer clinical medicine program in Tanzania. Generally, these findings suggest that a substantial proportion of raining institutions offering the diploma in clinical medicine in their current state, are ill-equipped to produce competent clinicians. These findings call for major investment to address the gaps in order to produce a competent health workforce. They also suggest need for regulatory bodies to strengthen monitoring and evaluation of HTIs to ensure compliance with the standards.

## Data Availability

The datasets generated and analysed during this study are not publicly available due to government restrictions on sharing data but are available from the Director, Sikika—Tanzania on reasonable request.
